# The Incidence of Infection Complicating Snakebites in Tropical Australia: Implications for Clinical Management and Antimicrobial Prophylaxis

**DOI:** 10.1155/2023/5812766

**Published:** 2023-10-12

**Authors:** Laura K. Smith, John Vardanega, Simon Smith, Julian White, Mark Little, Josh Hanson

**Affiliations:** ^1^Cairns Hospital, Cairns, Queensland, Australia; ^2^QML Pathology, Murarrie, Queensland, Australia; ^3^Women's and Children's Hospital, Adelaide, South Australia, Australia; ^4^NSW Poisons Information Service, Sydney NSW, Australia; ^5^The Kirby Institute, UNSW, Sydney, NSW, Australia

## Abstract

**Objective:**

To define the incidence of infection following snakebite in tropical Australia and the resulting implications for the routine prescription of prophylactic antibiotics.

**Methods:**

A retrospective study of all individuals presenting to Cairns Hospital, a tertiary referral hospital in tropical Australia, after a snakebite between December 2013 and October 2020.

**Results:**

There were 732 hospitalisations, 720 (98.4%) patients presented within 8 hours of the snakebite, and 29/732 (4.0%) were envenomated. Envenomated patients were more likely to receive empirical antibiotics than nonenvenomated patients (8/29 (27.6%) versus 14/703 (2.0%), *p* < 0.001), although this was frequently as a bundle of care for critically ill individuals. Superficial skin infection was diagnosed by clinicians in 6/732 (0.8%) patients during their hospitalisation; infection was diagnosed more commonly in envenomated than in nonenvenomated patients (3/29 (10.3%) versus 3/703 (0.4%), *p* = 0.001). All 3 envenomated individuals diagnosed with infection were believed to have taipan (genus *Oxyuranus*) bites. Five (83%) of the six patients diagnosed with infection had received empirical antibiotics at presentation; only 1/710 (0.1%) patients who received no antibiotics developed a (superficial) infection.

**Conclusion:**

Infection is a very uncommon complication of snakebite in tropical Australia. Individuals bitten by snakes in tropical Australia should not routinely receive antibiotic prophylaxis.

## 1. Introduction

The World Health Organization estimates that up to 5.4 million people are bitten by snakes every year and this results in up to 138,000 deaths [1]. Australia is home to some of the world's most venomous snakes, but successful public education campaigns have reduced the number of snakebites significantly. Despite this, there are still approximately 3000 snakebites annually in Australia, resulting in approximately 500 hospital admissions and, on average, 2 deaths [2].

Australian snakes are predominantly from the Elapid family with brown snakes (*Pseudonaja* spp.), tiger snakes (genus *Notechis*, *Tropidechis*, or *Hoplocephalus*), taipans (*Oxyuranus* spp.), death adders (*Acanthopis* spp.), and black snakes (*Pseudechis* spp.) the most medically important species. There has been significant recent evolution in the understanding of Australian snakebites and their management. A greater focus on prospective data collection, most notably the Australian Snakebite Project, has resulted in the establishment of state specific guidelines for management of snakebite and its complications, particularly envenomation. Envenomation syndromes vary by type of venom, and therefore snake, with clinically significant syndromes including venom-induced consumption coagulopathy, myotoxicity, neurotoxicity, acute kidney injury, and thrombotic microangiopathy [3].

Globally, there has been a natural, and appropriate, focus on life-threatening envenomation, but there has also been growing interest in the other complications of snakebite [4]. In many parts of the world, bacterial superinfection of snakebite is a common diagnosis (Table 1). One Taiwanese study found that wound infection complicated 23% of local *Naja atra* bites and resulted in significant morbidity: 46/117 patients required surgical intervention, which included 42 fasciotomies and 26 skin grafts [5]. A Brazilian randomised controlled trial reported that 44% of victims of *Bothrops* snakebites developed secondary infection if they were not prescribed prophylactic antibiotics [17]. Meanwhile, in a prospective study of patients admitted to a South African hospital requiring surgical debridement for skin and soft tissue necrosis following snakebite, 40/42 cases grew clinically significant bacteria and seven patients developed persisting functional impairment which included digital amputation, difficulty walking, and impaired elbow flexion [15].

These high reported rates of superinfection have led to some authors suggesting that routine antibiotic prophylaxis could be considered to prevent infection following snakebite envenomation particularly in necrotic wounds in resource-limited settings [4, 8, 18–21]. However, as many snake venoms cause significant local tissue injury and inflammation—a result of specific enzymes in the venom and the resulting local inflammatory response—the incremental contribution of bacterial secondary infection to venom-mediated tissue injury, and therefore utility of antibiotic therapy, is uncertain. This is important because routine empirical antibiotic therapy will also tend to increase the risk of antimicrobial-related complications, financial costs, and the development of antimicrobial resistance.

The venoms of common Australian elapids typically cause very little tissue injury [22], but the rate of secondary infection is incompletely described. This study was, therefore, performed to examine the rate of infection complicating snakebites in a tropical Australian referral hospital. The study sought to determine if there were any host, environmental, or health system factors associated with wound infection and whether infections occurred more commonly with any individual snake species. It was hoped that these data might inform decisions about the utility of empirical antibiotic therapy in people bitten by Australian snakes.

## 2. Methods

This retrospective study was performed at Cairns Hospital in tropical Far North Queensland. The hospital is a 531 bed tertiary referral centre that serves a population of approximately 290,000 people living across an area of 380,000 km^2^. Approximately 150,000 people live in the city of Cairns, the region's administrative hub, with the remainder living in nearby rural or more distant remote settings. It is considered a high volume snakebite centre, with rates of snakebite far greater than the country's southern capital cities [23]. Snakebites are managed at the hospital using national guidelines which are further informed by local clinical experience [22, 23].

Any admission to Cairns Hospital for snakebite (suspected or confirmed and based on clinical history and examination) with a completed discharge summary between December 12, 2013, and October 31, 2020, was eligible for inclusion in this study. This period was chosen as it coincided with the introduction of an electronic medical record in the hospital. Patients transferred to Cairns Hospital from peripheral hospitals with a diagnosis of snakebite were also eligible for the study.

International Statistical Classification of Diseases and Related Health Problems (ICD)-10 codes relating to venomous or nonvenomous snakebites, or contact with venomous and nonvenomous snakes, were used to identify potential study participants. Demographic, clinical, and laboratory data were collected from electronic patient medical records using a dedicated pro forma by two investigators (LS and JV) who met regularly to ensure the consistency of data collection.

Our study had a particular focus on the development of infection. Infection was defined as being present if attending clinicians documented any clinical signs consistent with this diagnosis, specifically erythema, warmth, pus, or blistering at the site of the bite(either during the initial emergency department presentation or during the subsequent hospital stay) and then commenced antimicrobial therapy on this basis. It was planned that any infection would then be further classified as superficial (cellulitis, lymphangitis, and subcutaneous abscess) or deep (septic arthritis, osteomyelitis, tenosynovitis, pyomyositis, and bacteremia). Laboratory investigations including microbiological investigations were reviewed. Any isolates that were considered contaminants by the attending microbiologist or that had a clear nosocomial source were excluded. Risk factors for infection were recorded using Australian national guidelines for the management of animal bites as a guide [24]. These included delay in seeking medical attention for ≥8 hours after the initial snakebite, the site of the bite, the presence of a foreign body or soiling, and the involvement of deeper tissues [25]. Complications of the bite including intensive care unit (ICU) admission or death were also recorded.

The medical record and laboratory results of each case were also examined to identify the presence of envenomation and the snake that was most likely responsible. The responsible snake was determined by reviewing the contemporary opinion of the clinical toxicologist involved in the case who, in turn, was informed by the clinical symptoms and signs, the geographic location of the snakebite, and the opinion of a herpetologist (if this was available).

### 2.1. Statistical Analysis

Data were deidentified, entered into an electronic database (supplementary file 1, Microsoft Excel 2016, Microsoft, Redmond, WA, USA), and analysed using statistical software (STATA version 14.2, StataCorp LLC, College Station, TX, USA). Univariate analysis was performed using the Kruskal–Wallis or Fisher's exact tests where appropriate. Multivariable analysis was performed using backwards stepwise logistic regression. Only variables that were significant in univariate analysis were included in the multivariable model. Trends over time were determined using Spearman's test with year as a continuous variable. Incidence was determined using population data from the Australian Bureau of Statistics.

### 2.2. Ethical Approval

The Far North Queensland Human Research Ethics Committee provided ethical approval for the study (HREC/2020/QCH/65939-1468QA). As the data were deidentified and retrospective, the committee waived the requirement for informed consent.

## 3. Results

A total of 734 cases were identified using clinical coding, but in two cases, it was not possible to confirm a snakebite in the medical record, leaving 732 episodes that were included in the analysis. These 732 snakebites occurred in 721 different individuals; 9 individuals were bitten on 2 occasions and 1 was bitten on three occasions. The patients' median (interquartile range (IQR) age at the time of presentation was 33 (19–52) years; 453/732 (62%) were male.

The mean (95% confidence interval (CI)) annual number of snakebite hospital presentations was 37.1 (31.7–42.4) per 100,000 population. There was no change in incidence over the study period (*p* for trend = 0.34) (supplementary Figure 1). Bites were most common in summer (228/732 (31%)) and least common in winter (117/732 (16%)) (supplementary Figure 2). Patients usually presented promptly to hospital with 720/732 (98.4%) presenting within 8 hours of the bite. Patients from rural and remote locations were as likely to present for medical attention within 8 hours as the patients from metropolitan areas (319/326 (98.5%) versus 399/406 (98.3%), *p*=1.0). Clinical and/or laboratory features consistent with envenomation were present in 29/732 (4%); after review by, or discussion with, a clinical toxicologist, all 29 received antivenom (Table 2).

### 3.1. Clinical and Laboratory Findings

Attending clinicians diagnosed only 6/732 (0.8%) patients with a wound infection; in all 6 cases, this was characterised as a superficial infection (Table 3). One was diagnosed on presentation and the other 5 were diagnosed with infection subsequently. In only one of these patients, a microbiological specimen was collected, a superficial swab that yielded no bacterial growth. Erythema developed in 3/6 (50%) within the first 24 hours, the remaining 3/6 developed erythema subsequently (2 during the hospital admission and the other on representation one day after hospital discharge). No patients with a diagnosis of infection had evidence of pus or blistering at any point. All six patients with a diagnosis of infection were bitten on a limb, compared to 711/726 (98%) of those without infection (*p* = 1.0). In only a single patient, there was a foreign body or soiling of the wound; this patient had 2 teeth from a nonelapid snake which were removed at surgery; the patient did not go on to develop infection. Only 2/732 (0.3%) patients had significant tissue injury: 1 had a crush injury while another had significant devitalised tissue, but neither of these patients went on to develop infection. There were 4/732 (0.6%) who required surgery at some point during their hospitalisation, but none of these patients developed infection.

The six cases diagnosed with infection had a higher white cell count, neutrophil count, and creatine kinase in the first 24 hours of their admission than the patients without infection (Table 4). Envenomated patients were more likely to be diagnosed with infection than nonenvenomated patient, and in multivariable analysis, only envenomation was independently associated with the diagnosis of infection (odds ratio (OR) (95% CI): 26.9 (5.2–139.8), *p* < 0.0001).

### 3.2. Association between Species of Snake and Subsequent Diagnosis of Infection

Out of the 6 cases that went on to develop infection, three cases were envenomated, and in all three cases, the toxicologist believed that a taipan (genus *Oxyuranus*) was responsible. Two were given polyvalent antivenom and one was given taipan specific antivenom. Patients envenomated by a taipan had a higher median (IQR) creatine kinase than patients envenomated by other venomous snakes (1345 (311–2443) units/L versus 207 (108–514) unit/L, *p*=0.003). In one case where infection was developed but the patient was not envenomated, the snake was identified by a zoologist as a Carpentaria whip snake (*Cryptophis boschmai*); in the two remaining nonenvenomated cases, the snake species was unknown (Table 2). No patients with confirmed bite by a brown snake (genus *Pseudonaja*, 4 patients), black snake (genus *Pseudechis*, 3 patients), tiger snake group (genus *Notechis, Tropidechis,* or *Hoplocephalus*, 3 patients), or death adder (genus *Acanthophis*, 1 patient) were diagnosed with infection.

### 3.3. Microbiology

Microbiology testing was performed infrequently. Of the 732 patients, 3 (0.4%) had a superficial swab collected: the one patient diagnosed with infection (described above) had no growth. A second patient's swab grew group C *Streptococcus*, while a third patient's swab grew mixed skin flora but neither of these 2 patients had a clinical diagnosis of infection. There were 5/732 (0.7%) who had blood cultures collected; 3 (60%) had no growth; the other two grew organisms were believed to represent contaminants: 1 grew *Corynebacterium* species, while the other, a patient admitted to ICU following a suspected taipan bite, had mixed growth of coagulase-negative *Staphylococcus* species and *Pseudoglutamicibacter cumminsii*. Clinically, this was thought to be related to line colonisation as opposed to wound infection from the snakebite, with repeated reviews of the bite site indicating an absence of infective features. None of the patients with a diagnosis of infection had blood cultures collected.

### 3.4. Use of Antibiotics

**Figure 1:**
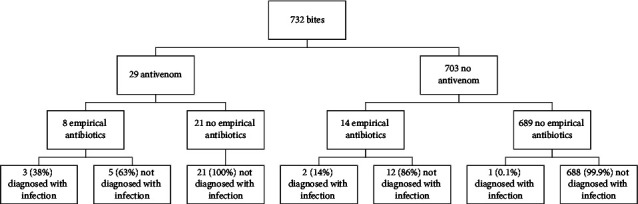
Impact of antibiotic prophylaxis on the subsequent development of infection. All patients in the cohort with clinical or laboratory evidence of envenomation received antivenom. Given the potential for significant adverse reactions to antivenom, individuals who have no clinical or laboratory evidence of envenomation did not receive antivenom.

There were 710/732 (97%) of patients who received no empirical antibiotic therapy; only 1/710 (0.1%) went on to be diagnosed with infection (Figure 1). Empiric antibiotics, when they were prescribed, were commonly given as a bundle of care: 5/14 (36%) patients admitted to ICU received antibiotics, compared with 17/718 (2%) of those who were not admitted to ICU (*p* < 0.0001). Envenomated patients were more likely to be given antibiotics than nonenvenomated patients (8/29 (28%) versus 14/703 (2%), *p* < 0.0001). Five of the 6 individuals diagnosed with infection received empirical antibiotics at presentation to hospital.

### 3.5. Patient Outcomes

None of the 6 patients that were diagnosed with infection required surgery, although one required ICU admission for management of their taipan envenomation. One patient was initially discharged, but was then readmitted with evolving swelling and worsening erythema at the bite site and spent a further 24 hours in hospital receiving intravenous flucloxacillin before being discharged home on oral flucloxacillin without subsequent complications. None of the 6 patients who developed infection died.

There were 14/732 (2%) admitted to ICU; all were envenomated. Of these 14 cases, 12 (86%) were believed to represent taipan bites and 2 of these patients died. The two other ICU admissions were believed to be due to a brown snake bite and a tiger snake group bite, respectively. The patient bitten by a brown snake died. None of the deaths were due to infections originating in the bite site.

## 4. Discussion

This study suggests that infection is an extremely rare complication of snakebite in tropical Australia. Almost 97% of 732 patients hospitalised with snakebite received no antibiotic therapy and only one went onto be diagnosed with a superficial infection. Envenomation was strongly associated with a subsequent diagnosis of infection, but it was notable that no infections were diagnosed in cases of envenomation by brown snakes, black snakes, tiger snake group, or death adders. In contrast, taipan envenomation was present in half of the cases where infection was diagnosed. As infection was diagnosed in half of the cases within 24 hours of the bite, and was associated with a rise in creatine kinase, it is possible that the local effect of the taipan venom resulted in clinical signs of inflammation at the bite site, which were diagnosed—possibly inaccurately—by attending clinicians as infection. While empirical antibiotic therapy has been recommended by some authors for the management of snakebites in other parts of the world [4, 6, 13], the extremely low rate of diagnosed infections in this series, a proportion of which are likely to represent local venom-mediated tissue injury rather than bacterial infection, suggests that, in Australia at least, it is not necessary to prescribe prophylactic antibiotic therapy for victims of snakebite.

The risk of infection in our cohort is likely to have been mitigated by the patients promptly seeking medical attention in Australia's well-resourced universal health system. Indeed, over 98% of people living in rural and remote settings were able to be reviewed within 8 hours of their initial presentation at a tertiary referral hospital, emphasising the effectiveness of the hub-and-spoke model of care in regional Australia [26, 27]. Despite the remote, tropical setting of the study, only a single case had a foreign body or soiling of the wound, suggesting that environmental contamination of the wound was a rare phenomenon.

While recognising that venomous snakes do not always inject venom when they bite, so-called “dry bites”, the very low rates of clinical envenomation suggest that a significant proportion of bites in this series were from nonvenomous snakes [28]. Nonvenomous snakes, such as pythons, are endemic in the area but are also common local pets. Only 0.4% of nonenvenomated patients developed an infection (superficial), replicating previous studies that suggest that antibiotic therapy is not required in nonvenomous snakebites [29, 30]. This finding also supports the hypothesis that the clinical findings suggesting infection in snakebite wounds may be secondary to inflammatory and myonecrotic effects of venom, rather than a true infection.

The venom of Australia's elapid snakes can result in a variety of haematological, neurological, and renal complications [22]. The clinical manifestations of envenomation vary according to the species of snake, although local complications, including skin necrosis and myonecrosis, are less common than in snakebites from other parts of the world. Bites of the Chinese cobra (*Naja atra*) and *Bothrops* species, for instance, are notable for their ability to cause local tissue damage. Chinese cobra venom can cause swelling, blisters, necrosis, and pain which can persist for many years [31]. Relevantly, these are the snakes that have been frequently highlighted in series where infection has been reported to be a common complication of snakebite. In contrast, other vipers, such as the green pit viper (*Trimeresurus macrops*) found in Thailand, have venom that causes less local tissue necrosis; individuals bitten by these snakes are less likely to be diagnosed with infection [7]. In our study, taipans were believed to be responsible for half of the bites in which infection was diagnosed by clinicians, and in all three of these taipan bites, individuals had a significantly elevated serum creatinine kinase. Taipan venom contains an enzyme called taipoxin, which is damaging to the striated muscle fibres causing muscle pain as well as a local inflammatory response, often manifesting as erythematous, but not necessarily infected, wounds [32].

Of course, venom-mediated tissue injury and bacterial infection are not necessarily mutually exclusive. Local tissue necrosis and wound breakdown may predispose to infection due to loss of the protective skin barrier. The lower rate of infection in our cohort may also be explained by differences between the fangs of Australian elapids and other snake species. The fangs of Australian elapids are relatively small and fixed, compared to the fangs of *Bothrops* species which are large and more likely to cause deeper puncture wounds, thereby increasing the risk of infection [25, 33].

The use of empiric antibiotics in snakebites remains controversial. The World Health Organization guidelines for the management of snakebites suggests the use of prophylactic antibiotics in necrotic wounds or if nonsterile instruments have been used in wound care [34]. Other reviews have suggested that if bite wounds have been incised or obviously contaminated, or if there is evidence of necrosis rather than just local bruising, immediate empirical treatment with an affordable broad-spectrum antibiotic is justified [4]. However, many studies that suggest that empirical antibiotics may have utility are based on bacteriological studies of snake's oral flora [19, 20]. The importance of a wound's clinical appearance was emphasised in a South African study which reported no adverse outcomes if no antibiotics were withheld in wounds without necrosis or an abscess [35]. Even studies that identify bacteria in necrotic wounds do not necessarily establish that the isolated bacteria have a causative pathophysiological role [36]. Furthermore, in well conducted prospective, randomised controlled trials empirical antibiotic therapy does not prevent the development of infection, even in necrotic wounds in resource-limited settings [17, 37, 38].

Australian guidelines make no specific mention of antibiotic prophylaxis in snakebites, although they recommend that, in general, animal bite wounds should be cleaned and irrigated, and amoxicillin-clavulanate prescribed either as presumptive or empiric therapy for in patients with high-risk wounds, with intravenous piperacillin-tazobactam recommended as an alternative if deeper tissues are involved [24]. However, our study suggests that empirical antibiotic therapy of snakebites in tropical Australia is likely to have little benefit, while also increasing financial costs and the risk of antimicrobial-related complications. These concerns are significant in a region where the mean annual incidence of snakebites during the study period was at least 37.1 (31.7–42.4) per 100,000 population, compared with an estimated annual incidence in Brazil of 13.4 per 100,000 population [39] and in India of 18.2 per 100,000 population [40].

This retrospective study has many limitations. It is possible that some of the cases included in the study were not snakebites, but rather bites from other animals or insects or a traumatic injury mischaracterised as a snakebite. This emphasises the importance of the history and examination and highlights the challenges faced by both patients and clinicians in snakebite identification. The reported local incidence of snakebites is almost certainly an underestimation, as not every snakebite in the region is transferred to, or admitted to, the Cairns Hospital. The rarity of infection in the cohort limits the reliability of multivariable analysis which uses infection as the dependent variable, although the findings of the analysis in this cohort accord with our hypothesis that venom-mediated tissue injury may be misdiagnosed as infection. It is also important to acknowledge that this study was carried out in a developed country and tertiary hospital and examined only Australian snakes. Our findings and recommendations should therefore be interpreted with caution by clinicians in other geographical locations.

## 5. Conclusions

The rate of infection amongst patients in tropical Australia who are bitten by snakes, whether they are envenomated or not, is very low and patients usually have excellent outcomes. The use of empiric antibiotics in snakebite is a complex issue globally, and there is no universally applicable strategy. Important considerations include the health care setting and access to care, local microbiology, and antibiotic resistance, as well as the snake species and the presence of envenomation. However, our study suggests that individuals bitten by Australian snakes do not routinely require empirical prophylactic antibiotics.

## Figures and Tables

**Table 1:** Comparison of infection rates complicating snakebites that have been reported in series from different countries.

Reference	Country	Snake species	Infection rate (%)	Comments
[5]	Taiwan	*Protobothrops mucrosquamatus* and *Viridovipera stejnegeri*	22.5	All patients received antivenom
[6]	Taiwan	*Trimeresurus mucrosquamatus* (41.1%) *T. stejnegeri* (21.6%), and cobra (10.8%)	25.5	*Morganella* species in 14%
[7]	Thailand	Green pit viper (*Trimeresurus* sp.)	6.5	Haemorrhagic bleb only risk factor for infection
[8]	China	*G. brevicaudus* and *D. acutus*	25.1	Excluded readmissions
[9]	Brazil	*Bothrops* sp.	15.1	Only included children
[10]	Brazil	*Bothrops* sp.	40	Included all patients
[11]	Brazil	*Bothrops* sp.	23.3	Included all patients
[12]	Colombia	*Bothrops asper*	30.8	
[13]	Martinique	*Bothrops lanceolatus*	12	Risk of infection increased with severity of envenomation
[14]	United States	Rattlesnake	0.98	
[15]	South Africa	Mozambique spitting cobra (*Naja mossambica*)	25.6	Only included patients requiring surgical debridement
[16]	Ghana	*Echis ocellatus*	35.3	

**Table 2:** Selected laboratory findings (within the first 24 hours of admission) and clinical course of the 29 cases of envenomation in the series.

Case	Age, gender	Snake^a^	White cell count^b^	Neutrophil count^c^	Creatinine kinase^d^	Empirical antibiotics on admission	Diagnosed with infection	ICU admission	Outcome
1	50, male	Taipan	11.6	9.4	1730	Yes	Yes	Yes	Survived
2	16, male	Taipan	17.9	15.6	1880	Yes	Yes	No	Survived
3	39, male	Taipan	27.1	24.6	316	Yes	Yes	No	Survived
4	59, female	Taipan	6.8	5.2	11300	No	No	Yes	Died
5	16, male	Taipan	5.8	4.8	286	Yes	No	Yes	Survived
6	77, male	Taipan	25.2	13.5	779	No	No	Yes	Died
7	33, male	Taipan	14.4	12	33300	Yes	No	Yes	Survived
8	15, male	Taipan	24.5	20.2	1160	No	No	Yes	Survived
9	39, male	Taipan	11.9	9.9	2220	Yes	No	Yes	Survived
10	46, male	Taipan	18	15.6	294	No	No	Yes	Survived
11	58, male	Taipan	13.1	11.4	560	No	No	Yes	Survived
12	69, male	Taipan	17.4	15.7	3480	No	No	Yes	Survived
13	67, male	Taipan	13.6	12	1530	No	No	Yes	Survived
14	31, female	Taipan	14.9	12.1	463	No	No	Yes	Survived
15	47, male	Taipan	16.6	12.3	163	No	No	No	Survived
16	48, male	Taipan	23.6	20.9	3110	No	No	No	Survived
17	67, male	Taipan	16.4	14.9	1840	No	No	No	Survived
18	62, female	Taipan	14.6	13.4	243	Yes	No	No	Survived
19	40, male	Brown	15.7	13.6	2610	Yes	No	Yes	Died
20	23, male	Brown	10.8	8.8	514	No	No	No	Survived
21	55, male	Brown	11.8	9.2	241	No	No	No	Survived
22	51, male	Brown	36.1	31.6	1170	No	No	No	Survived
23	69, female	Tiger group	14	11.4	108	No	No	Yes	Survived
24	23, female	Tiger group	10.6	7.5	207	No	No	No	Survived
25	23, male	Tiger group	11.6	8.2	90	No	No	No	Survived
26	27, female	Black	19.2	17.8	143	No	No	No	Survived
27	44, female	Black	14.9	11.8	74	No	No	No	Survived
28	60, male	Black	6.3	6.3	218	No	No	No	Survived
29	42, male	Death adder	18.1	15.3	172	No	No	No	Survived

^a^Based on contemporaneous toxicologist opinion informed by clinical and laboratory findings, geographical location of the bite, and pathological envenomation syndrome. ^b^Reference range: 4.0–11.0 × 10^9^/L. ^c^Reference range: 2.0–8.0 × 10^9^/L. ^d^Reference range: 46–171 U/L.

**Table 3:** Characteristics of the six patients in the cohort who were diagnosed with infection following the snakebite.

Case	Age, gender	Delayed presentation^a^	Received empirical antibiotics at presentation	Clinical signs of infection at presentation	Signs of infection at the bite site^b^	Other signs of infection	Envenomation^c^	Snake	Antibiotics	Microbiological isolates
1	39, male	No	Yes	No	Warmth and erythema	Inguinal lymphadenopathy pain	Yes: 1 ampoule polyvalent antivenom	Taipan^d^ (*Oxyuranus* genus)	Doxycycline (oral), metronidazole (oral)	No
2	16, male	No	Yes	No	Erythema	Pain	Yes: 1 ampoule taipan antivenom	Taipan^d^ (*Oxyuranus* genus)	Flucloxacillin (oral)	No
3	50, male	Yes	Yes	Yes	Erythema	No	Yes: 2 ampoules polyvalent antivenom	Taipan^d^ (*Oxyuranus* genus)	Piperacillin and tazobactam	No^e^
4	4, female	No	No	No	Erythema	Local swelling, inguinal lymphadenopathy	No	Unknown	Cephalexin (oral)^f^	No
5	50, female	No	Yes	No	Warmth and erythema	Local swelling	No	Unknown	Flucloxacillin	No
6	45, female	No	Yes	No	Warmth and erythema	Local swelling, pain, axillary lymphadenopathy	No	Carpentaria whip snake *Cryptophis boschmai*^g^	Flucloxacillin	No

^a^Greater than 8 hours after bite. ^b^At any point during the episode. ^c^Contemporary opinion of clinical toxicologist. ^d^Based on opinion of clinical toxicologist informed by clinical and laboratory findings, geographical location of the bite, and pathological envenomation syndrome. ^e^Superficial swab sent, no growth. ^f^Represented with worsening pain and erythema same day of discharge, kept overnight for intravenous flucloxacillin and discharged on oral flucloxacillin. No reported complications. ^g^Confirmed by zoologist.

**Table 4:** Comparison of patients who were diagnosed with infection during their hospitalisation and those who were not.

Variable	Diagnosed with infection, *n* = 6	No infection, *n* = 726	*p* ^a^
Age (years)	42 (13–50)	33 (19–52)	0.82
Male gender	3 (50%)	450 (62%)	0.68
Lowest haemoglobin^b^ (g/L)	134 (124–144)	137 (126–147)	0.60
Highest white cell count (×10^9^/L)^b^	11.2 (9.2–20.2)	8.3 (6.9–9.9)	0.009
Highest neutrophil count (×10^9^/L)^b^	8.3 (5.0–17.8)	4.6 (3.6–6.0)	0.008
Lowest platelets (×10^9^/L)^b^	243 (189–339)	228 (196–265)	0.52
Highest aPTT (seconds)^b^	34 (28–38)	30 (27–33)	0.24
Lowest fibrinogen (g/L)^b^	2.8 (2.3–3.3)	2.5 (2.1–3.0)	0.57
Highest INR^b^	1.1 (1.1–1.2)	1.1 (1.0–1.1)	0.23
Highest creatinine kinase (units/L)^b^	483 (154–1768)	153 (106–240)	0.02
Highest creatinine (*µ*mol/L)^b^	68 (47–98)	73 (60–87)	0.73
Highest CRP (mg/L)^b c^	34	1.0 (1.0–9.7)	0.08
Erythema^b^	3 (50%)	25 (3%)	0.001
Pus^b^	0	0	—
Blistering^b^	0	1 (0.1%)	1.0
Antivenom administered	3 (50%)	26 (4%)	0.001
Taipan bite	3 (50%)	15 (2%)	<0.0001
Brown snakebite	0	4 (0.6%)	1.0
Black snakebite	0	3 (0.4%)	1.0
Tiger bite	0	3 (0.4%)	1.0
Death adder bite	0	1 (0.1%)	1.0
Bite on limbs	6 (100%)	711 (98%)	1.0

All numbers represent absolute number (%) or median (interquartile range). aPTT: activated partial thromboplastin clotting time; INR: international normalised ratio; CRP: C-reactive protein. ^a^Unadjusted analysis presented. ^b^In the first 24 hours of hospitalisation. ^c^Only 1 patient who was diagnosed with infection had a C-reactive protein measured in the first 24 hour.

## Data Availability

The data used to support the findings of this study are provided in the supplementary file.

## Supplementary Materials

Supplementary Materials Supplementary Figure 1: Annual incidence of snakebite presentations per 100,000 population during the study period. Supplementary Figure 2: Month of the snakebite in this cohort. In the southern hemisphere, spring is September to November inclusive, summer is December to February inclusive, autumn is March to May inclusive, and winter is June to August inclusive. In far North Queensland, the monsoonal wet season occurs between November and May.
