# Supplementary figures and images for: The Incidence of Infection Complicating Snakebites in Tropical Australia: Implications for Clinical Management and Antimicrobial Prophylaxis
Source: J Trop Med. 2023 Oct 12;2023:5812766. doi: 10.1155/2023/5812766 (PMC10586896; doi:10.1155/2023/5812766)

## Slide 1
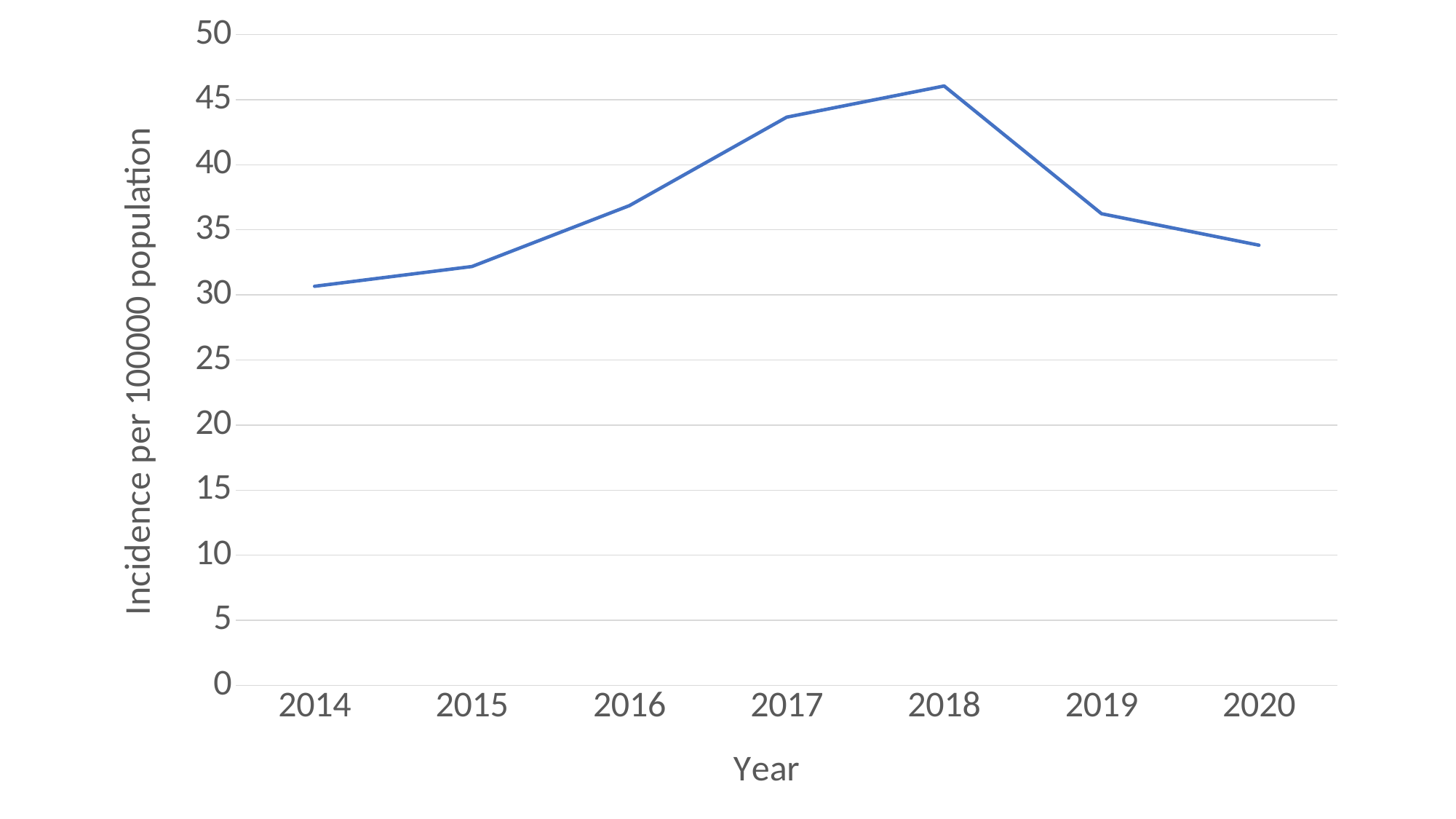

### Chart
| Category | Incidence (per 100000 population) |
|---|---|
| 2014 | 30.66241773163814 |
| 2015 | 32.183642031113266 |
| 2016 | 36.85692713421289 |
| 2017 | 43.653089254596885 |
| 2018 | 46.047474594799795 |
| 2019 | 36.23756511437482 |
| 2020 | 33.819810602848825 |

Supplement: Supplementary Materials — Supplementary Figure 1: Annual incidence of snakebite presentations per 100,000 population during the study period. Supplementary Figure 2: Month of the snakebite in this cohort. In the southern hemisphere, spring is September to November inclusive, summer is December to February inclusive, autumn is March to May inclusive, and winter is June to August inclusive. In far North Queensland, the monsoonal wet season occurs between November and May. [file 5812766.f1.zip › Supplementary figure 1.pptx]

## Slide 1
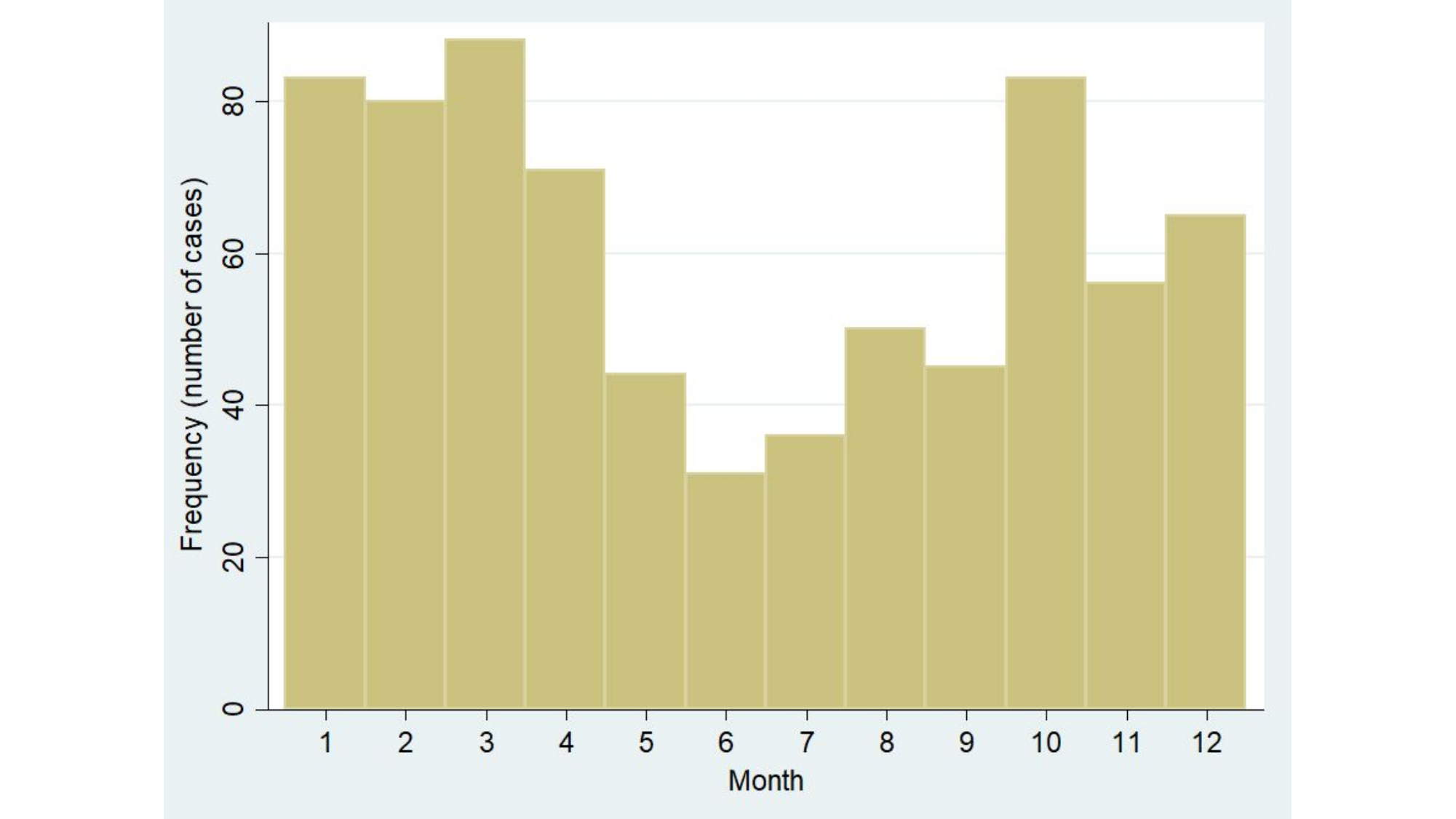

Supplement: Supplementary Materials — Supplementary Figure 1: Annual incidence of snakebite presentations per 100,000 population during the study period. Supplementary Figure 2: Month of the snakebite in this cohort. In the southern hemisphere, spring is September to November inclusive, summer is December to February inclusive, autumn is March to May inclusive, and winter is June to August inclusive. In far North Queensland, the monsoonal wet season occurs between November and May. [file 5812766.f1.zip › Supplementary figure 2.pptx]
